# Understanding demand for, and feasibility of, centre-based child-care for poor urban households: a mixed methods study in Dhaka, Bangladesh

**DOI:** 10.1186/s12889-020-09891-z

**Published:** 2020-12-10

**Authors:** H. Elsey, F. Fieroze, R. A. Shawon, S. Nasreen, J. P. Hicks, M. Das, R. Huque, I. Hirano, H. J. Wallace, M. Saidur

**Affiliations:** 1 Department of Health Sciences, University of York, Seebohm Rowntree Building, York, YO10 5DD UK; 2 ARK Foundation, Suite no C3, C4. House no. 6, Road no 109, Gulshan 2, Dhaka, 1212 Bangladesh; 3 Department of Public Health Sciences, Centre for Injury Prevention and Research Bangladesh (CIPRB), House # B-162, Road # 23, New DOHS, Mohakhali, Dhaka, 1206 Bangladesh; 4 Nuffield Centre for International Health and Development, University of Leeds, Room 10.31, Level 10, Worsley Building, Leeds, LS2 9NL UK; 5 Nuffield Centre for International Health and Development, University of Leeds, Room 1029, Level 10, Worsley Building, Leeds, LS2 9NL UK; 6 Nuffield Centre for International Health and Development, University of Leeds, Worsley Building, Leeds, LS2 9NL UK; 7 Present address: Japanese International Cooperation Agency, 5-25 Nibancho, Chiyoda City, Tokyo, 102-0084 Japan; 8 School of Medicine, University of Notre Dame Australia, 19 Mouat St, Fremantle, Western Australia 6959 Australia

## Abstract

**Background:**

Centre-based child-care has potential to provide multiple health and development benefits to children, families and societies. With rapid urbanisation, increasing numbers of low-income women work with reduced support from extended family, leaving a child-care vacuum in many low- and middle-income countries. We aimed to understand perceptions of, and demand for, centre-based child-care in Dhaka, Bangladesh among poor, urban households, and test the feasibility of delivering sustainable centre-based child-care.

**Methods:**

We used sequential mixed methods including a household survey (*n* = 222) and qualitative interviews with care-givers (*n* = 16), community leaders (*n* = 5) and policy-makers (*n* = 5). We co-produced and piloted a centre-based child-care model over ten-months, documenting implementation. A co-design focus group with mothers, parents’ meetings, and qualitative interviews with child-care centre users (*n* = 5), non-users (*n* = 3), ex-users (*n* = 3) and staff (2) were used to refine the model and identify implementation issues.

**Results:**

We found 24% (95% CI: 16,37%) of care-givers reported turning-down paid work due to lack of child-care and 84% (95% CI:74, 91%) reported wishing to use centre-based child-care and were willing to pay up to 283 Takka (~$3.30) per month. Adjusted odds of reported need for child-care among slum households were 3.8 times those of non-slum households (95% CI: 1.4, 10). Implementation highlighted that poor households needed free child-care with food provided, presenting feasibility challenges. Meta-inference across quantitative and qualitative findings identified the impact of the urban environment on child-care through long working hours, low social capital and fears for child safety. These influences interacted with religious and social norms resulting in caution in using centre-based child-care despite evident need.

**Conclusion:**

Sustainable provision of centre-based care that focuses on early childhood development requires subsidy and careful design sensitive to the working lives of poor families, particularly women and must respond to the dynamics of the urban environment and community values. We recommend increased research and policy focus on the evaluation and scale-up of quality centre-based child-care, emphasising early-childhood development, to support low-income working families in urban areas.

## Background

Centre-based child-care has the potential to provide multiple benefits to children, families and societies [1] yet, has received limited attention in policy and research particularly in low and middle-income countries (LMIC) [2]. With rapid urbanisation in LMICs [3], the neighbourhoods where many children now grow up are fundamentally different from the rural communities of previous generations. Nowhere is this clearer than in Dhaka, Bangladesh, where the city population grows by 3 to 4% every year and as of 2019, an estimated 18 million live within the greater Dhaka area [4].

This rapid and uncontrolled urbanisation is coupled with significant societal change as more women join the formal and informal workforce [5]. These changes are experienced disproportionately by women from poorer households; in Dhaka, 33.4% of women living in slums work full-time compared to 14.6% in the rest of the city [6]. These families who must frequently work long hours, with limited availability of extended family, face a childcare vacuum. There is limited information from the informal sector, but within the formal sector, 39% of women must mind their child at work [7]. This situation is mirrored in other LMICs where the lack of adequate childcare results in higher than expected numbers of children enrolling in year one of primary school [8].

The challenges of providing a safe, healthy environment for young children in urban neighbourhoods are reflected in the poor health outcomes experienced by children living in slums in LMICs [9]. Within Dhaka, intra-urban differences are stark, with poor nutrition and high prevalence of infectious diseases resulting in 50% of children with stunted growth in slum areas compared to 33% in non-slum areas [6]. The impacts of the limited support for young children can also be seen in educational attainment with only 65% of children from slum communities attending primary school, compared to 84% outside the slums [6]. This evidence supports the body of knowledge from high income countries (HICs) that a child’s place of residence influences their development and health [10].

The provision of quality centre-based child-care has the potential to counteract some of the negative influences of poor urban neighbourhoods. There is evidence of social and economic benefits of centre-based child-care on women’s participation in the labour force in LMICs [11–13], with increases in parental employment, particularly of mothers, providing indirect benefits to the child through increased household income and improved nutrition [14]. There have been few high-quality studies of the impacts of centre-based care on children’s health and development have been conducted in LMICs [2]; what limited evidence there is, indicates improvements in cognitive [14] and socio-emotive development [15]. Evidence from both HICs [16] and LMICs [17] indicates that cheap, but poor-quality centre-based child-care may worsen early childhood development (ECD) outcomes. No evidence of nutritional benefits have yet been found [15], but further studies are underway in Africa [18] and Asia [19]. Evidence from Bangladesh, where drowning is a major cause of childhood mortality [20] indicates that rural, community-based child-care centres can significantly reduce childhood drowning and all-cause mortality, making centre-based child-care a highly cost-effective intervention in the prevention of injuries [20]. In order to find viable policy and practical solutions to the child-care vacuum in urban areas, we aimed to understand how the characteristics of urban neighbourhoods and the perceptions of their residents influence the demand for child-care and the feasibility of delivering centre-based care in Dhaka, Bangladesh.

### Study setting

We purposively selected a mixed-income ward in the south of Dhaka with nine Mahallas [smaller neighbourhood for religious and community functions] and an elected ward commissioner. The ward is densely populated covering an area of only 2.87 km^2^ yet inhabited by around 93,000 households [21] and characterised by small industries, particularly plastic and metal recycling, manufacturing of metal, leather products, as well as shoe and dress-making. Poor regulation of working environments results in hazardous conditions for workers and those living near-by, particularly children. The ward consists of permanent and non-permanent housing often located side-by-side. Better-off and poor households are frequently found on different floors in the same building, with small industries often occupying the ground floor. Roads are narrow and frequently impassable except by foot, bike or rickshaw.

## Methods

### Study design

We used a sequential mixed methods design [22, 23] comprising both quantitative and qualitative methods in sequence to deepen and broaden the understanding of centre-based child-care demand and to explore the feasibility of delivering centre-based child-care. Table 1 presents the methods and samples used in the two phases of the study.

**Table 1:** The phases and methods used in the study

Target population		Method	Purpose	Planned sample	Actual sample
Phase 1: understanding perceptions and demand	Households with a child under-5	Questionnaire	To assess demand for centre-based child-care and current child-care practices	200 households	222 households
	Community leaders	Qualitative Semi-Structured Interviews (SSIs)	To understand perceptions of centre-based child-care	5 SSIs	5
	Mothers, fathers and guardians	Qualitative SSIs	To understand perceptions of and demand for centre-based child-care	8 SSIs with those wanting to, 8 SSIs with those unwilling to use child-care centres	*With those wanting to use child-care centres:* 9 SSIs with mothers, 3 SSIs with fathers 2 SSIs with grandmothers *With those unwilling to use child-care centres:* 2 SSIs with fathers. Unable to recruit mothers, guardians unwilling to use centre-based care.
	Policy-makers and ECD experts	Qualitative SSIs	To understand the context of ECD and centre-based care in Bangladesh	5 SSIs	5
Phase 2: understanding implementation and feasibility	Mothers, fathers and guardians	Co-design focus groups (FGs)	To gain feedback on the planned model and inform the detailed specification	2 FGs: 1 with slum and 1 with non-slum parents/ guardians.	1 co-design FG of 8 mothers from slum households, willing to use a child-care. Unable to recruit FG of non-slum households.
	Users, non-users and centre staff	Qualitative SSIs Centre users’ meetings Monthly enrolment data	To understand experiences of using the child-care centre and to adapt the model to meet the needs of low-income families	Users, non-users and staff of the centre	3 users’ meetings 10-months of enrolment data SSIs: 2 staff, 5 mothers still using centre, 3 mothers no-longer using the centre, 3 non-users who despite initial interest did not take up a place.
	Households survey participants	Follow-up questionnaire 6 months after phase 1	To identify the proportion of respondents traceable at 6 months	222 households	159 households traced

### Phase 1 understanding perceptions and demand for centre-based child-care

#### Household survey

Our mixed-methods study began with a household survey to: i) quantify demand for centre-based child-care and identify associations with socio-demographic variables, current child-care practices, needs and attitudes to centre-based child-care; ii) identify parents’ pre-requisites and recommendations for design of the child-care centre.

For the quantitative aspect of the survey we wanted to estimate proportions for categorical question response categories at a 10 percentage point or lower margin of error (95% confidence interval (CI) width). Assuming a design effect of 2 due to our clustered sampling design we estimated that we required a sample size of 193 households to achieve this level of precision. We used a two-stage cluster design. First, one Mahalla was selected by simple random selection out of the 9 Mahallas in the ward. In the selected Mahalla there were 19 lanes each with approximately 500 households and each lane was considered as one cluster. According to the demography of Bangladesh, 10% of households have children under-five [24]. Children under-5 were the focus of this study, as those over 5 years are eligible for pre-primary education in Bangladesh [25]. Thus, in the second stage of sampling we selected 7 clusters via simple random sampling and interviewed all the households with any children under-five. Three data collectors and one supervisor, all Bangladeshi, collected data. The questionnaire was pre-tested among slum and non-slum households resulting in the re-phrasing of several questions (see supplementary materials - questionnaire). Each household was asked to identify a household member with detailed knowledge of the children and child-care practices in the home to complete the questionnaire in a face-to- face interview. Table S1 in the supplementary materials presents the contents of the final questionnaire and relevant respondents.

#### Qualitative methods

As part of our sequential design, we used our survey results to purposively select mothers, fathers and care-givers of children under-5 from both slum and non-slum households [26], who had stated in their questionnaire that they were willing or unwilling to use centre-based child-care. We used semi-structured interviews to build an in-depth understanding of child-care practices and beliefs about centre-based care and interviewed community leaders to understand wider social norms. All interviews were conducted by two Bangladeshi researchers, both with training and significant experience in qualitative methods. Guides were used in the interviews (, with researchers adapting these to probe in-depth on issues of relevance raised by participants. Interviews were audio-recorded and reflective notes were taken. All data were transcribed into English by transcribers fully trained and briefed on the project. This phase of data collection took place between August 2017 to May 2018.

### Phase 2: understanding implementation and feasibility

#### Co-design and implementation of centre-based care

**Fig. 1:**
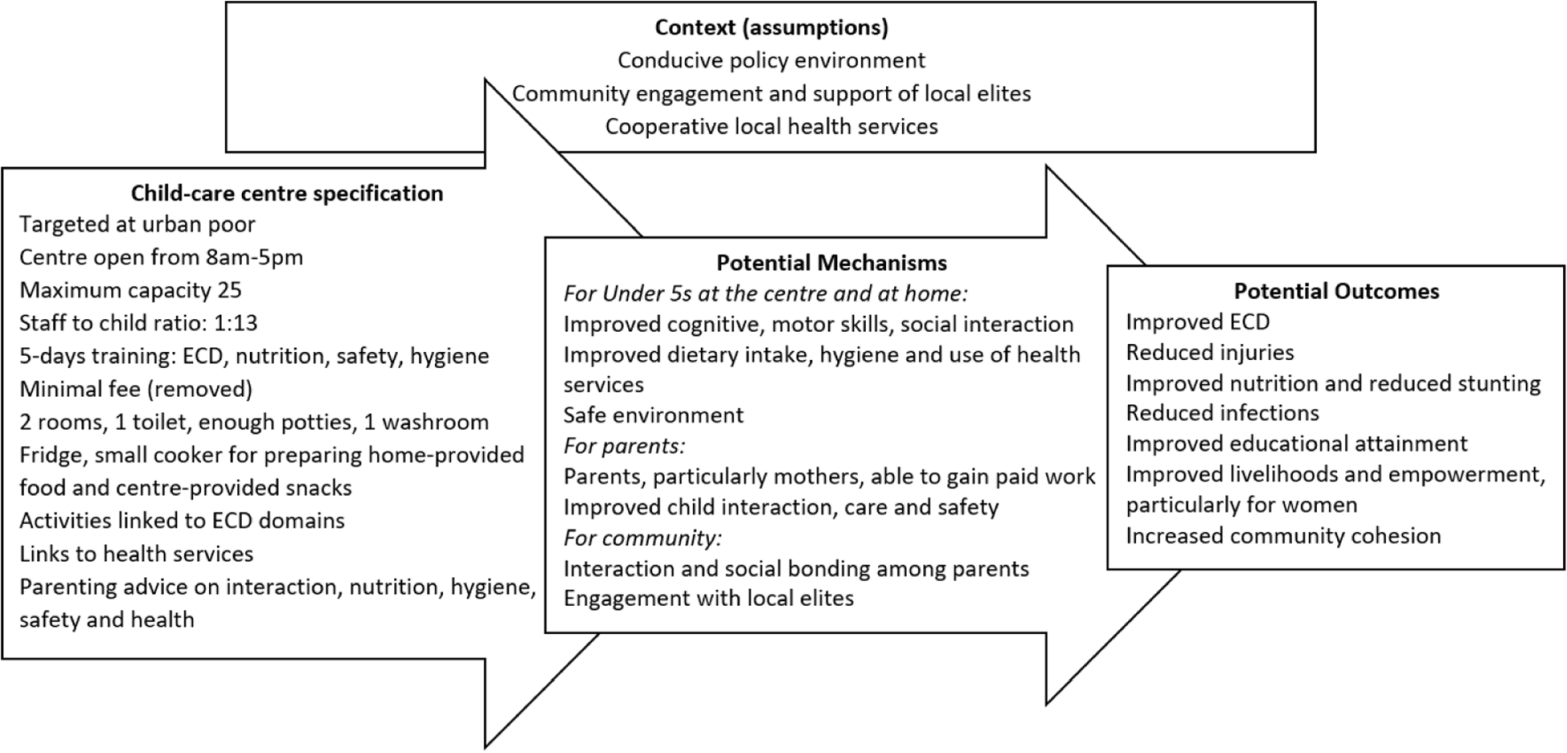
Theory of change and specification of the co-designed child-care centre model

We developed the initial child-care centre model based on the structures and curriculum developed by Centre for Injury Prevention and Research (CIPRB, lead partners in this study) for their rural community-based child-care programme [27] and UNICEF guidelines [28]. We aimed to adapt this model based on analysis of the Phase 1 qualitative data and household survey, co-design focus groups and detailed discussions with our steering group. Our steering group consisted of experts from Bangladesh ECD Network, representatives from national and international non-governmental organisations running child-care centres in the community and in workplaces, Ministry of Health and Ministry of Women and Child Affairs. The group developed the theory of change (see Fig. 1), updated the team on policy developments and provided detailed feedback on the design of the centre. Once designed, the centre was implemented in the selected ward for a 10-month period (January to October 2018). During this time, we aimed to use the cycles of participatory action research (PAR) of plan, act, observe, reflect [29] with monthly meetings of centre users and staff to discuss experiences, challenges and try out possible improvements to the model. Training on PAR was provided to the team from the two research organisations in Dhaka; one organisation (CIPRB) led the facilitation of the PAR sessions (RS) and researchers from the ARK Foundation observed and recorded the process. At the end of the implementation period, qualitative interviews were planned with centre staff, parents who had used the centre, ex-users that had left the centre and parents who after initial interest, had not taken up a place.

### Follow-up of survey respondents

Given the transient nature of many urban residents, particularly poorer households, we wanted to assess the feasibility of following-up respondents at 6 months. By using contact details supplied in the first survey, respondents were contacted by phone, or if phone contact was unsuccessful, they were visited at home.

### Data analysis

#### Quantitative

The survey data were summarised descriptively and estimates of proportions (categorical variables) and means (continuous variables) were presented with 95% confidence intervals. We then used multiple logistic regression to explore the association between a range of socio-demographic variables (see table footnotes for full details) and selected outcomes, with inferences about the direction, size and statistical significance (5% significance level) of associations based on adjusted odds ratios (AOR) and their associated 95% confidence intervals and two-sided *p*-values. All analyses were done in Stata version 14 (StataCorp. 2015. Stata Statistical Software: Release 14. College Station, TX: StataCorp LP.). We accounted for the clustered survey design in all our inferential analysis via Stata’s *svyset* commands using Taylor linearisation methods [30]. Where outcome and/or covariate data were missing we did complete case analysis.

#### Qualitative

Qualitative data from all participants and methods were analysed following the stages specified in framework approach [31, 32], this facilitated multiple analysts to work together, often at a distance. Room was allowed for the inductive emergence of themes. The final thematic framework was developed by three researchers coding the same three transcripts blindly, discussing their individual coding and then agreeing on a final coding frame. The results from the questionnaire survey were also used to guide the analysis, in particular, exploration of issues facing slum households and parents’ use of other institutions for child-care. Qualitative data from the parents’ meetings and the final round of interviews with centre staff, users and ex-users were coded within the same coding frame, with new codes emerging and expanding the understanding of the pre-existing themes. NVIVO version 11 was used to manage qualitative data analysis.

#### Integration of the quantitative and qualitative elements

As described above, the analysis of the survey informed the sampling of participants for qualitative interviews and focus groups. Further, identification of the needs and preferences of parents and care-givers identified in the survey and qualitative analysis informed the development of the child-care model and subsequent interviews with users, non-users and ex-user. Finally, to gain insights beyond the separate analysis of the quantitative and qualitative data, the research team integrated the findings by identifying themes (meta-inferences) from across all quantitative and qualitative data [23].

## Results

### Participant characteristics

The first household survey was conducted between July and August 2017. Of the 239 households approached, 222 households consented to completing the questionnaire (response rate 92.9%). Characteristics of the survey population are given in Table 2. Seventy-two per-cent of households were classified as ‘slum’ or ‘non-slum’ based on the UNHABITAT 5-point definition of slum households [26]. Full details of the participants in the qualitative interviews conducted in phase 1 with care-givers (*n* = 16), community leaders (*n* = 5) and in phase 2, with child-care centre users (*n* = 5), non-users (*n* = 3), ex-users (*n* = 3) and staff (2) are presented in Table S2 (supplementary materials). The final specification of the centre following the co-design process and then reshaped during implementation can be found in table S3 (supplementary materials).

**Table 2:** Characteristics of survey population

	Frequency	%
Child age (years)		
1 to < 3.5	129/222	58
3.5 to < 5	93/222	42
Child sex		
Male	114/222	51
Female	108/222	49
Primary care-giver role		
Mother	192/211	86
Father	2/211	1
Sister	2/211	1
Grandmother	13/211	6
Other	2/211	1
Primary care-giver education status		
Illiterate	41/222	18
Literate	181/222	82
Primary care-giver occupation		
‘Housewife’ - not working outside household	182/220	83
Skilled worker	19/220	9
Unskilled worker	19/220	9
Missing	2/222	
Household status^a^		
Slum	160/222	72
Non-slum	62/222	28
Duration living in the area		
Less than a year	13/215	6
1–2 years	20/215	9
3 years or more	182/215	85
Missing	7/222	3

Missing values are excluded from frequencies and percentages. ^a^Based on UNHABITAT definition (UNHABITAT, 2007)

Through the meta-inference analysis, we identified five overarching themes which highlight how social, religious and gender norms interact with the working lives of poor urban families and neighbourhood characteristics to influence demand for, and feasibility to provide ECD-focused centre-based care. The first three themes shed light on perceptions of centre-based child-care: (i) child benefits, (ii) social capital and trust in an urban environment, (iii) family first. The fourth theme (iv) making a living, contributes to the understanding of demand for centre-based child-care and the final theme (v) focuses on feasibility. The meta-inferences under each theme and the methods from which they are derived are summarised in Table S5 (supplementary materials) and presented below.

#### Perceptions: child benefits

Within our theory of change (see Fig. 1), we identified the potential for positive ECD, health and safety outcomes for children. Within our findings we identified how these elements were perceived across participant groups and methods.

*Early childhood development (ECD):* There was a disconnect between parents and policy-makers in terms of understanding and priority given to ECD. Interviews with parents and community members highlighted how their priority was the academic aspects of ECD, with a focus on literacy and numeracy. Policy makers felt parents and the wider community rarely appreciated the wider social, emotional and cognitive aspects of ECD:

*“Because in our country, we see that the meaning of children’s care to most of our mothers is only providing the children’s food and bath. They don’t have any idea about other factors besides that.” (Policy-maker 03 SSI, ECD expert)*

Parents frequently expressed concerns about the care they were able to provide due to the demands of their working lives in the city, and in particular, how this would impact on their child’s education:

*“At the moment, care is just about giving a child proper food, giving them a proper bath, making sure they sleep on time…. Besides that, they must get a proper education. I started teaching my other children when they were 2 years old. But I could not teach her even a simple rhyme and she’s already 3 years…. I just can't give her much time. I leave home at 9am and get back at 10pm.” (M-016 SSI: mother, slum-household)*

Grandmothers were identified as regular secondary care-givers in the interviews and 6% of primary care-givers in the survey. However, the care they provided frequently did not meet the parents’ child-care aspirations, particularly in terms of educating the child:

*“Definitely, it’s not how I want it to be. The thing is, my mother is an uneducated, older woman so neither can she teach him anything, nor does she even try to teach him anything. She does her own things all day, she just feeds him and gives him bath, that’s all.” (M019 SSI: mother, non-slum, school assistant)*

There was, however, considerable variation in how our participants interpreted ‘education’. The discussion among participants in the co-design focus group illustrated how some mothers clearly gave a high priority to learning specific subjects, while others focused on learning rhymes. While they mentioned how children love to play, none explicitly mentioned playing with their own children:

*PI:“I think there should be three teachers. One should teach Bangla, one English and the other maths.*

*P2: No, no sister, it is not possible. You have to understand, the children will learn the alphabet, rhymes, no separate teacher is necessary for that.*

*P4: Exactly, the children will study different subjects on different days… they don’t have to study all the subjects every day.*

*P6: Right, the children should be taught along with their playing games. If they are only made to study, they will be unwilling to go to the centre anymore.*

(Co-design focus group discussion)

The survey suggested that despite pre-primary not starting until 5 years old, several under-5 s were attending school, with 10% (95% CI: 5–19%), already enrolled in a school or Madrasah and 14% (95% CI: 6–30%) planning to enrol soon.

*Religious education*: The desire to provide a good religious upbringing for their children was a common aspiration among parents. Within the ward there were privately run Quomi Madrasahs and Alia Madrasahs which provide Islamic education alongside the government curriculum [33] and Furkania or Hafizia Madrasah where children of 3 to 4 years learn to recite the Holy Quran [34]. We found the Madrasah education system was highly valued, particularly among fathers, as it delivered religious instruction whilst also offering a free child-care service often with food provided.

*“There are many Mosques and Madrasahs in this area – I like these institutions. I enjoy the scene of the little children going to Mosque and Madrasah every day.” (F-256 SSI: father, slum-household, factory worker) and “I will try my best to make the boy Maulana [graduate of Islamic education].” (F-172 SSI: father, slum-household, factory worker).*

For some, centre-based care was seen as detrimental to religious education and child development:

*“Initially, most people would think very negatively about these types of places [child-care centres]. They think the children who stay there will watch something they shouldn’t watch; and their name will be erased from the list of Muslims. They consider it to be an ‘orphanage’. It makes me feel bad.” (F063 SSI: father, slum household, shop- keeper).*

*Health and safety:* In our survey, we found 24% (95% CI: 15–36) of children had had an injury and 69% (95% CI: 59–78) an illness in the last 6 months. We found no significant associations with childhood injuries; however, we found a statistically significant, modest positive association with being female versus male and childhood illness (adjusted odds ratio (AOR: 1.9, [95% CI: 1.2, 3.1]) and a significant, modest positive association with those care-givers who said they needed a secondary care-giver compared to those who did not (AOR: 1.5, [95% CI: 1.1, 2.2]). Conditions within the urban neighbourhood, coupled with the challenges of supervising children, may help to explain the frequency of injuries:

*“There is no open space for children to play in this area, there are no fields. Children have to play in the street or the lane. The roads in this area are very narrow and uneven. Kids have real difficulty playing safely.” (F-256 SSI: father, slum household, factory worker)*

#### Perceptions: social capital and trust in an urban environment

Social aspects of the urban environment also influenced child-care practices and perceptions of centre-based care. The findings from across all methods highlighted low levels of trust and fearful attitudes towards others living within the neighbourhood, with serious concerns for the safety and behaviour of their children. A common concern was the influence of ‘bad’ friends:

*“These children love to play with others and end up learning abusive language and bad behaviour.” (CL IMAM SSI: Male, community leader: religious leader)*

*“But some mothers can only go to work if they keep their kids at home alone or with elderly grandparents and then the problems arise. After some time, the kids will just go outside and play with bad friends.” (M-172 SSI: mother, slum-household, business)*

Concerns about the safety of the neighbourhood and lack of extended family led to difficult child-care decisions for parents. Interestingly, and possibly reflecting low levels of reciprocity and trust within the community, none of the participants mentioned leaving their children with neighbours, even when this meant not allowing children out of the house for fear of their safety.

*“No, this place is not safe. Often children go missing. No one knows who anyone is, where they are going and what they are doing. … Often I hear the announcement on the microphone that a child has gone missing.” (M018 SSI: mother, non-slum, housewife)*

The community leaders explicitly mentioned these concerns as something that would undermine enrolment in the centre-based child-care centre:

*“Suppose, I gave my baby there [the child-care centre] and he or she was smuggled. It is better to play in the streets than that… Such fear really affects the people.” (CL IMAM SSI: Male, community leader: religious leader)*

These concerns were reflected in the questionnaire results with the most common reason for not wanting to enrol in a child-care centre was concern that they would be supervised by an unknown person (46% [95% CI: 28–65%]).

Phase 2 of our study found that despite these underlying concerns, users built up their trust as they became more familiar with centre staff and practices. As the mother below explains, her husband’s attitude changed after her child had attended the centre for several months:

*“My husband first said ‘no’ because the centre is unknown. He said, ‘Do not admit her, keep her close’. I said: “now everyone’s child is there, and they do not face any problems, so my child won’t have any problems either. My child will study properly. My husband did not say anything then. He goes there sometimes, just to watch them. Now he likes it too.” (User03 SSI: mother)*

Interviews with community leaders highlighted social divisions within the neighbourhood, with the needs of tenant migrants, or ‘floating-people’ who have no voting rights and little voice within the community, rarely taken into consideration:

“*I am not sure whether this area requires a child-care centre….There are some floating people in this area who rent houses and are mostly labourers. They could be day labourers … they live from hand to mouth; they may need a centre. For the permanent residents, these mothers don’t have to work or be day labourers, which could cause them to keep their child with someone else or, in an institution. I am working as a public representative for last two years and no one has come to me with this problem to seek a solution (CLWC SSI: Male, community leader: elected official)*

Another factor undermining social capital was the high mobility of urban residents. Our second survey found 41% (95% CI: 31–52%) of families had moved house in the 6 months since the base-line survey with 13% (95% CI: 8–20%) moving outside Dhaka 10% (95% CI: 4–22%) within the ward, 73% (95% CI: 59–83%) within the Mahalla and 4% (95% CI: 1–14%) elsewhere in Dhaka (Table 3).

**Table 3:** Phase 2 household survey: Follow-up of base-line survey participants at 6 months

Variable	Frequency/total	% (95% CI)
**Previous respondents who could be traced by either mobile phone or household visit**		
Yes	159/222	72 (59–82)
No	63/222	28 (18–41)
**Agreed to participate in a questionnaire interview about their situation, child, centre-based child-care needs.**		
Yes	125/159	79 (72–84)
No	30/159	19 (13–27)
Don’t know	4/159	3 (1–5)
Missing	63/222	28

#### Perceptions: family first

Despite the apparent need for child-care, centre-based care was still seen as a last resort, only to be used if care was not available from a family member. The continuation of traditional perceptions that women, either as a mother, grandmother or aunty should be the main care-giver was evident. Centre-based child-care was the last resort, once support from female relatives and older siblings had been exhausted, this was particularly evident in interviews with male community leaders and fathers:

*“If the sister-in-law or the mother-in-law of the wider family is unwilling to look after the child, then parents would have no other choice but to keep their child in a child-care centre. But, this could result in a bad relationship with their family.” (CL LP SSI: male, community-leader elected official)*

Despite these traditional norms of women as the ideal care-givers, the interviews highlighted how with more women working outside the home, some fathers were taking an active role in child-care. Despite the potential for changing gender norms, none of the participants explicitly mentioned the role of fathers in providing child-care.

#### Demand: work and childcare

We found a high level of demand with 84% (95% CI: 74–91%) of survey respondents willing to pay to enrol their under-5 child in centre-based child-care. Furthermore, 24% (95% CI: 16–37%) of care-givers, the majority of whom (86%) were mothers, reported previously turning down paid work due to lack of child-care. We found a significant, large, positive association with wishing to enrol in centre-based care and being from a slum versus non-slum household (AOR: 3.8 [95% CI: 1.4, 10]) (Table 4).

**Table 4:** Demand for centre-based child-care and relationships with child, care-giver and household characteristics

	Prepared to enrol in centre-based child-care			Prepared to pay for centre-based child-care			Prepared to pay extra to subsidise centre-based child-care for children from low-income families		
	n	% (95% CI)	AOR (95% CI); *p*-value	N	% (95% CI)	AOR (95% CI); *p*-value	n	% (95% CI)	AOR (95% CI); *p*-value
All households	136/215	63 (48–76)	NA	187/222	84 (74–91)	NA	92/169	54% (35–73)	NA
Slum/non-slum status of household									
Non-slum	27/59	46 (24–69)	Ref	46/62	74 (52–89)	Ref	30/44	68% (31–91)	Ref
Slum	109/156	70 (56–81)	3.8 (1.4, 10); 0.016	141/160	88 (80–93)	2 (0.8, 4.9); 0.1	62/125	50% (32–67)	0.6 (0.3, 1.1); 0.08
Age (child)									
3.5 to < 5	43/89	48 (31–66)	Ref	73/93	78 (70–85)	Ref	44/70	63% (37–83)	Ref
1 to < 3.5	93/126	74 (61–84)	2.9 (1.4, 6.2); 0.013	114/129	88 (70–96)	1.9 (0.5, 6.8); 0.25	48/99	48% (31–67)	0.6 (0.2, 1.1); 0.11
Sex (child)									
Female	63/105	60 (40–77)	Ref	91/108	84 (75–91)	Ref	47/83	57% (34–77)	Ref
Male	73/110	66 (54–76)	1.7 (0.9, 3.3); 0.1	96/114	84 (68–93)	1.4 (0.9, 5.4); 0.56	45/86	52% (34–70)	0.9 (0.5, 1.7); 0.68
Need secondary care-giver									
No	87/148	59 (43–73)	Ref	124/152	82 (71–89)	Ref	60/111	54% (40–68)	Ref
Yes	46/63	73 (52–87)	2.4 (1.1, 5.2); 0.032	60/66	91 (80–96)	2.2 (0.9, 5.4); 0.07	30/55	55% (24–82)	1 (0.3, 4); 0.94
Primary care-giver (PCG) working									
No	112/177	63 (47–77%)	Ref	155/184	84 (71–92)	Ref	77/136	57% (36–75%)	Ref
Yes	24/38	63 (45–78)	0.6 (0.3, 1.2); 0.11	32/38	84 (72–92)	0.6 (0.2, 1.9); 0.35	15/33	45% (29–63%)	0.7 (0.3, 1.5); 0.27
PCG ever missed work due to lack of childcare									
No	95/153	62 (48–74)	Ref	131/158	83 (73–90)	Ref	68/118	58% (40–74)	Ref
Yes	39/53	74 (52–88)	1.1 (0.4, 2.8); 0.83	48/53	91 (81–96)	1.4 (0.7, 2.9); 0.26	17/44	39% (14–70)	0.5 (0.2, 1.7); 0.23
PCG education status									
Literate	108/174	66% (48–80)	Ref	151/181	83 (7–91)	Ref	78/135	58% (34–78)	Ref
Illiterate	28/41	62 (48–74)	0.8 (0.3, 2.1); 0.66	36/41	88 (58–97)	1 (0.2, 5.9); 0.97	14/34	41% (31–52)	0.5 (0.2, 1.1); 0.08

Missing cases are excluded from frequencies and percentages. Confidence intervals for percentages are logit transformed and account for the clustered survey design. AOR = adjusted odds ratio. Ref = reference group for categorical variable effect comparison. For each outcome the adjusted odds ratios, their 95% confidence intervals and associated *p*-values are obtained from a logistic regression model (that accounts for the clustered survey design) including all listed covariates, excluding missing cases (complete cases for models: prepared to enrol in centre-based child-care = 195/222, prepared to pay for centre-based child-care = 210/222, prepared to pay extra to subsidise centre-based child-care for low socio-economic status children = 161/222)

Interviews with community leaders helped to explain the increase in poorer, slum households as established home-owners moved out of the area, renting out their houses to factory workers. The shifting population had led to changes in family structures, with fewer large, extended families. For working parents this lack of extended family led to significant child-care challenges.

*“So, the husband and wife of most of the families have to work outside the home… suppose they have three children and the elder child is eight or ten years old, then the parents really depend on that elder child, leaving their younger child under their responsibility.” (CLBM SSII, male, community-leader: business).*

A common strategy for working mothers, particularly single mothers, was to take their young children to work with them. Only one of the parents interviewed, who worked in a local school, had child-care provision at work. For those with no such provision, attempting to work whilst caring for the child was seen as detrimental to both the child and their work:

*“I have to take my child with me when I’m selling the cloths. When my daughter was 5 months old, from that age I used to keep her on my lap wherever I go. And even when I go to work , I had to take my child with me, this is difficult and painful for me.” (M 172 SSI: mother, slum household)*

Such strategies were clearly challenging and could lead to job-loss, as one woman who used our child-care centre, reflected:

*“I cannot do anything properly when my child is at home. Now my daughter is going to the centre regularly and I don’t have any problems with my work. Before if I went to work, I had to take her with me. She would make mischief and people at work got angry and they scolded her. I quit a job angrily because of that; a good job in a factory.” (Child-care centre user 03)*

Even when adult family members were available to take care of children, parents still faced challenges when either the child or the carer became sick or unable to provide care:

*“But if her grandparents become sick or if they go to village for some reasons, then it becomes very difficult for me to take care of her. I face huge problems at work, I have to take leave and stay at home, I cannot go to work then.” (FGP3 FGD: Mother, factory-worker)*

The qualitative interviews highlighted how, while some participants identified as housewives (83%, see Table 1), they were still attempting to earn some income at home by making sweets, handicrafts and sewing. Several of the women who used our centre were able to increase their income-generating work at home. The category of ‘housewife’ within the survey may well have underestimated the proportion of women attempting to earn an income without working outside the home.

#### Feasibility: fees, food, hours and engagement

We adapted the initial rural centre-based child-care model following results of the phase one survey, the advice of the steering group and the co-design focus group with eight mothers. The specification of the resulting centre model is provided in Fig. 1 and in table S3 in the supplementary materials. Specific adaptation to the rural model included long open hours (8 am-5 pm) to cover parents’ working day, the facilities needed to store and prepare food and increased provision for children under 3.5 years such as sufficient potties and specific training of providers.

Throughout the implementation of the model in phase 2, we planned to hold monthly user-group meetings with parents to enable re-design of centre features in line with their needs and suggestions. Despite our attempts to arrange monthly meetings, the long-hours worked, multiple caring and household commitments (e.g. household gas supply was erratic, meaning mothers had to stay at home to cook if gas availability coincided with the time of the user-meeting) meant that we were only able to hold three meetings during the year of implementation (Supplementary materials: table S4).

Despite the demand for the centre being highest among slum households (AOR 3.8 [95%CI 1.4–10]), survey respondents specified they could pay a mean amount of 218 takka (95% CI: takka 187–249) per month or ~ $2.55 US dollars (95% CI: $2.19–$2.92) with no food provided and 283 takka (95% CI: takka 185–382) per month or ~ $3.30 (95% CI: $2.17–$4.47) with food. Initially a minimal fee of 200 takka/month (~$2.40) was charged when the centre opened, however, enrolment was low in the early months of implementation and feedback from the user-group highlighted how even paying a minimal fee of 200 takka was seen as too much by many families. To boost enrolment among poorer families, the fee was removed.

We often found contradictions between the findings from the different methods (questionnaire, qualitative interviews and users’ group meetings) on parents’ specifications for the centre-based child-care centre. Most fundamentally, while the initial survey suggested a high demand for centre-based child-care, we found that throughout the 10-month implementation period we were not able to fill the centre to its capacity of 25 children. Enrolment varied per month, starting slowly with only 8 children and reaching a maximum of 22 children by the fifth month. Over the 10 months, a total of 35 children used the centre, the majority of whom (63%) came from slum households [26]. Seasonal changes and religious events influenced demand:

*“We are experiencing drop-out because it is the month of Ramadan. Only eleven or twelve children come here every day. It is decreasing because most of the people are tenants here. Some are returning to the village again some are moving back home.” (SSI Centre staff member)*

Finding ways to provide nutritious food within the limited space and budget of the centre was a key challenge. The initial survey indicated that 92% (95% CI: 83–96) were willing to provide food. However, this led to challenges within the centre with some children having more, and tastier, food than others. Staff and the user-group felt this undermined the spirit of equity within the centre and there was concern for nutritional adequacy for poorer children but also concern that any fees would deter these families. It was agreed that the only way to continue to provide care for children of poorer households was the provision of snacks (fruit, eggs, bread) at no extra cost. This placed a further challenge to the sustainability of the centre. Further details of the issues raised in the user-group can be found in supplementary table S4.

**Fig. 2:**
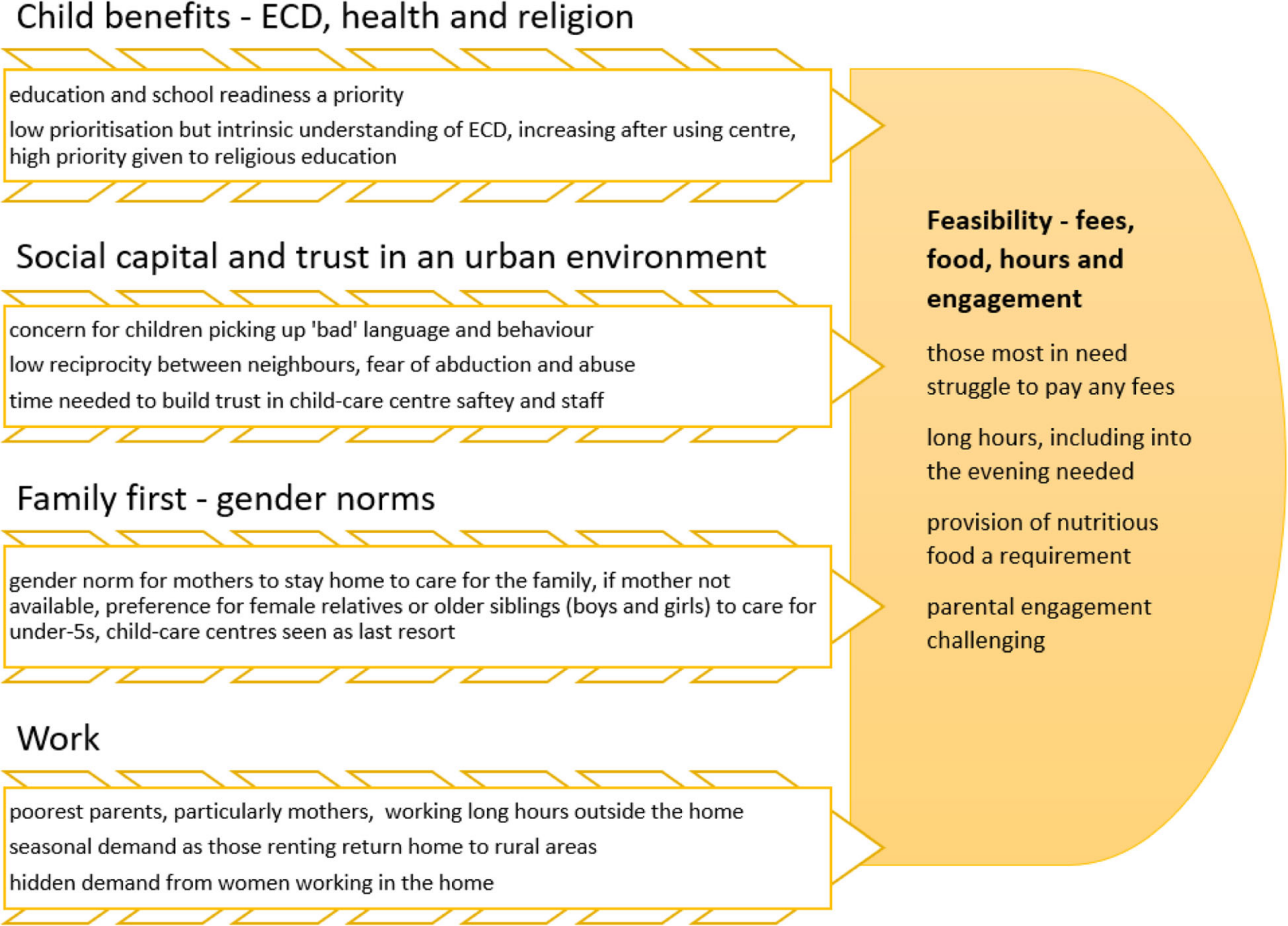
Summary of factors driving demand for, and feasibility of, child-care centres

As summarised in Fig. 2, the findings from both phases of the study shed light on why, even with no fees, sustaining enrolment at the centre was challenging. The inter-relationships between the urban social and physical environment, perceptions of appropriate providers of child-care based on gender-norms and value placed on education and religion, rather than holistic ECD, influenced parents’ willingness to use centre-based care.

## Discussion

We found a complex picture of demand for child-care centres which influenced the feasibility of provision of a sustainable model with the urban context. Initial findings from the household survey showed high levels of demand – as indicated by willingness to pay – of 84% (95% CI: 74–91%), with almost four times the odds 3.8 (95% CI: 1.4, 10) of slum households wishing to enrol than non-slum. Despite this demand, uptake of places at the centre was limited. The World Bank reports a number of studies [1, 35] highlighting that low use of child-care services is not due to a lack of demand, but rather demand is curtailed due to the lack of services at the right price, times and of good quality. In addition to these factors, our study points to several other influences on the uptake of child-care centre places. These include factors relating to the local physical and social environment, the norms and values of the community as well as the characteristics of families themselves in terms of their working lives, income and family structures. The combination of these factors fits well with Macintyre et al’s [36] explanation of the influence of place on health through the interaction of contextual (local physical and social environment), compositional (characteristics of individuals concentrated in particular places) and collective (socio-cultural) factors [36].

A key contextual feature of the urban neighbourhood that drove the limited uptake of centre-based care in our study was the lack of trust and low social-capital. Parents were acutely concerned about the safety of their children. Such concerns, and the media reporting of accidents and mistreatment of children have been found to discourage child-care centre use in Thailand [37]. In contrast, quality, centre-based care which includes child safeguarding policies, practices and provider training have been identified as increasing use of centre-based care in Latin America and the Caribbean [38]. Parents′ belief that their neighbourhood is safe has been found to be positively associated with children’s social–emotional development and general health [39].

Our findings highlight how the composition of the community with parents working long hours and high levels of mobility, both permanent and seasonal, influenced demand. These compositional aspects in turn influence the collective, socio-cultural influences on demand for day-care. For example, the limited capacity for parental engagement in shaping the day-care centre was driven by parents’ limited available time. Previous studies have highlighted challenges of community engagement in urban areas and the negative impact this can have on health outcomes [40, 41]. Finding ways to engage low-income parents in urban areas requires particular attention, may take time and require one-to-one as well as group meetings held outside the working day and week. Given that 55% of children globally now live in cities [42], further studies to understand how social cohesion can be strengthened with the urban context is vital [43].

The shared norms and traditions or, in Macintyre et al.’s (2002) conceptualisation, collective aspects helped to explain the reluctance of families to use centre-based care. Women’s participation in the labour force was a necessity for poor households; yet traditional attitudes that women should stay at home caring for children prevailed among community leaders, with the second-best option being other family members, particularly siblings and grandmothers, providing child-care. The high value placed on religious instruction and academic learning further reduced demand for ECD-focused centre-based care. This chimes with previous studies in Asia [44] and Bangladesh [25] which have found low value placed on socio-emotional caregiving by parents and is borne out in analysis of survey data from across 28 LMICs [45]. While the number of users of our centre was small, we did see increasing appreciation for the ECD approach among parents. Other studies indicate that supporting parents to understand the value of stimulating and emotionally responsive care may ultimately increase demand for quality centre-based care offering ECD [46].

Ultimately, we found that running a centre focused on quality ECD and also providing food, appropriate space and hygiene facilities that was also affordable and covered the long working days of urban households was unfeasible to deliver sustainably without external funding. This differs from rural areas in Bangladesh where non-governmental organisation-run child-care centres that are only required to provide care for short hours (i.e. 2 or 3 h per day) have been found to be sustainable [47]. The recent passing of the Child Care Act (2020) to regulate centres and ECD Policy of 2013, which provides a framework to deliver quality ECD for under-5 s, are positive policy developments in Bangladesh. A key priority for future research is the development and evaluation of models of centre-based child-care that can be delivered at scale for low-income households in urban areas [48].

Our study has several limitations. Firstly, we focused on only one ward in Dhaka. Factors driving demand may be different in urban areas with different characteristics, particularly given the complex interplay of compositional, contextual and collective factors. We had planned focus groups with both slum and non-slum participants, but were unable to recruit any non-slum residents. This may have limited the voice of this group within the co-design process reducing our understanding of willingness of better-off households to share centre-care with children from slum households and to subsidise provision. Another limitation of our study is the short period of time (10 months) that we were able to implement our centre, and the limited parental engagement we were able to facilitate. With a longer implementation period, we might have been more successful at engaging parents. Our use of a mix of methods in a sequential manner over 2 years is a strength of the study allowing development of meta-inferences across different methods (table S5) to shed light on the complex influences on demand for centre-based day-care in a poor, urban neighbourhood.

## Conclusion

There is a high demand for centre-based child-care in urban areas, particularly among poor households. The working lives of poor parents, particularly mothers, changes to family structures and lack of a safe physical and social environment undermine provision of a healthy environment for child development. Despite significant need, sustainable provision of centre-based care requires significant subsidy and careful design sensitive to the working lives of poor families, particularly women, the local physical and social environment and community norms and values. We recommend an increased focus on centre-based care within policy and research to enable the provision at-scale of quality ECD-focused childcare centres accessible to urban poor households.

## Supplementary Information

**Additional file 1: Table S1**. Questionnaire design: concepts and respondents. **Table S2**. Characteristics of the qualitative sample of parents, care-givers, community leaders, centre staff, ECD experts and policy makers. **Table S3**. Key features of urban childcare centre model following co-design and shaping during 10 months of implementation. **Table S4**. Child-care centre user group meetings. **Table S5**. The development of meta-inference across the methods used in all phases of the study.

**Additional file 2.** Anchal Childcare centre demand questionnaire English.

## Data Availability

The quantitative data from the household surveys is available for analysis by other research teams in the University of Leeds data repository and can be found here: 10.5518/902 The qualitative data is available for further analysis by other researchers, but we request research teams to be in contact with the corresponding author (helen.elsey@york.ac.uk) to ensure that the context of the qualitative data is properly understood and informs any subsequent analysis.

## Abbreviations

AOR: Adjusted Odds Ratio
ARK Foundation: Advancement through Research and Knowledge (ARK) Foundation
CIPRB: Centre for Injury Prevention and Research Bangladesh
CI: Confidence Interval
ECD: Early Childhood Development
FG: Focus Group
HIC: High Income Country
LMIC: Low and Middle-Income Country
NA: Not applicable
PAR: Participatory Action Research
PCG: Primary Care-Giver
SSI: Semi-Structured Interview
Ref: Reference
UNICEF: United Nations Children’s Fund
UNHABITAT: United Nations Human Settlements Programme
