# Supplementary material for: Understanding demand for, and feasibility of, centre-based child-care for poor urban households: a mixed methods study in Dhaka, Bangladesh
Source: BMC Public Health. 2020 Dec 10;20:1899. doi: 10.1186/s12889-020-09891-z (PMC7727228; doi:10.1186/s12889-020-09891-z)
Supplement: Supplementary file 1 — Additional file 1: Table S1. Questionnaire design: concepts and respondents. Table S2. Characteristics of the qualitative sample of parents, care-givers, community leaders, centre staff, ECD experts and policy makers. Table S3. Key features of urban childcare centre model following co-design and shaping during 10 months of implementation. Table S4. Child-care centre user group meetings. Table S5. The development of meta-inference across the methods used in all phases of the study. [file 12889_2020_9891_MOESM1_ESM.docx]

**Supplementary Materials**

**Table S1: Questionnaire design: concepts and respondents**

| **Respondent** | **Demographic details** | **Child-care arrangements** | **Health** | **Opinions on**  **centre-based child-care** |
| --- | --- | --- | --- | --- |
| **Purpose** | **To understand characteristics in relation to child-care needs** | **To understand existing child-care arrangements and needs** | **To inform future choice of outcome measures** | **To understand demand and inform co-design of child-care centre.** |
| Primary care giver | Age; sex; religion; education; occupation; monthly income;  slum or non-slum as per UNHABITAT (UNHABITAT 2007) definition | Primary care-giver’s working status; need for a child-care; current use of centre-based child-care; provision of care for others’ children | Under-5s’ injuries in last 6 months and  illnesses in last one month | Perceived need for child-care; wish to enrol and willingness to pay for centre-based child-care; specifications for centre-based child-care. |
| 1 to <3.5 years | Age, sex |  | Height and weight |  |
| >3.5 to 5 years | Age, sex |  | Height and weight |  |

**Table S2: Characteristics of the qualitative sample of parents, care-givers, community leaders, centre staff, ECD experts and policy makers**

| **Individual interviews with caregivers purposively sampled from survey participants** | | | | | | | | | | | |
| --- | --- | --- | --- | --- | --- | --- | --- | --- | --- | --- | --- |
| ID | Child-care role | | | | Slum/  Non slum | Willing or not  to use centre-based child-care | Gender | | Ages of children 5 years or below | Occupation | |
| M-016 | Mother of 3 children. Grown-up daughter and son (11-15years). Daughter (age range: 3-5 years old) is looked after by grandmother (who recently had a stroke) and son when home from school. | | | | Slum | willing | Female  Age range:  31-40years | | Daughter  Age range: 3-5 years old | Garment Worker  Her husband is a factory worker and rickshaw puller | |
| M-019 | Mother of 1 son, (age range: 3-5 years old) looked after by aunt or left alone at home. | | | | Non-slum | willing | Female,  Age range: 21-30 years | | Son  Age range: 3-5 years old | School assistant  Husband works in a ‘curd’ shop | |
| M-022 | Mother of one son (age range: 3-5 years old). She works at home so can feed her son during the day, and her mother-in-law helps to look after the boy while she works. | | | | Non-slum | willing | Female  Age range: 21-30 years | | Son,  Age range: 3-5 years old | In business with her brother in law as a seamstress.  Husband works in a garment factory as an ‘iron man’ | |
| M-063 | Mother of 2 children: older daughter (age range 6-10 years) stays with her grandmother in the rural area attending a madrassa during the day. Daughter (age range: 3-5 years old) lives with mum and looked after during the day by her father-in-law | | | | Slum | willing | Female  Age range: 21-30 years | | 1 daughter  Age range: 3-5 years old | Working 12 hour-days in a lock factory. Her husband works in a cosmetic factory. | |
| M-108 | Mother of one son, (age range 0-3 years old) taken care of by her sister-in-law during the day. She leaves house at 8.30 and returns 6pm. | | | | Non-slum | willing | Female  Age range: 31- 40 years old | | 1 son  Age range: Under 3 years old | Government worker (auditor), husband works for a private business | |
| M-172 | Mother of 3 children: oldest son (age range 11-15 years old) is at school in the rural area, son (age range 11-15 years old) in madrassa , daughter son (age range 3-5 years old) in Islamia school | | | | Slum | willing | Female  Age range: 31- 40 years old | | 1 daughter  Age range: 3-5 years old | Runs her own business selling clothes. Husband is unemployed | |
| M-216 | Mother of 2 children: son (age range 11-15 years old) at school and daughter (age range 3-5 years old), stays with employer’s household during the day | | | | Non-slum | willing | Female  Age range: 21- 30 years old | | 1 daughter  Age range: Under 3 years old | Auto-machine factory worker. Husband is a driver working 10am-10pm | |
| M-256 | Mother with 2 daughters. She works at home as a tailor. Daughter (age range 11-15 years old) has autism, young daughter (age range under 5 years old). Mother-in-law helps look after both children while she is working. | | | | Slum | willing | Female  Age range: 31- 40 years old | | 1 daughter  Age range: Under 3 years old | Tailor working from home, husband works as a care-taker | |
| M-259 | Mother with 3 children: oldest son (age range 5-10 years old) and daughter (age range 5-10 years old)study in the madrassa during the day. Son (age range under 3 years old) is looked after by mother-in-law in the neighbouring apartment. | | | | Slum | willing | Female  Age range: 21- 30 years old | | 1 son  Age range: Under 3 years old | Garment factory worker working 10am to 10pm. Husband also a garment factory worker | |
| F-063 | Father of two daughters: eldest daughter (age range 5-10 years old) stays with grandparents (in-laws) in the rural village and attends madrassa during the day. Youngest (age range 3-5 years old) looked after by paternal grandparents who live with them. | | | | Slum | Willing | Male  Age range: 31- 40 years old | | 1 daughter  Age range: Under 3 years old | Sales assistant in a shop. His wife also works outside the home (unspecified) | |
| F-172 | Father of 2 sons and 1 daughter: eldest son (age range 10-15 years old) is studying in the village, another son (age range 11-15 years old) in the madrassa and he looks after the daughter (age range: under 5 years old) in the morning and the mother looks after in the afternoon. | | | | Slum | Willing | Male  Age range: 31 -40 years old | | 1 daughter  Age range: 3-5 years old | Works in a restaurant for 3pm to 12pm. Wife makes and sells clothes. | |
| F-256 | Father of 2 daughters, one (age range 11-15 years old) with a disability and one (age range: under 5 years old) looked after by the mother | | | | Slum | Willing | Male  Age range: 31- 40 years old | | 1 daughter  Age range: 3-5 years old | Working at a plastic sorting factory from 8am to 4pm  Wife makes clothes at home | |
| F-259 | Father of 3 – husband of M-259  Son (age range 5-10 years) and daughter (age range 5-10 years) study in the madrassa during the day and son (age range under 5 years) is looked after by mother-in-law in the neighbouring apartment. | | | | Slum | Not willing | Male  Age range: 21- 30 years old | | 1 son  Age range: Under 3 years old | Garment factory worker and wife also works full-time in garment factory. | |
| F-271 | Father of 2 daughters: (age ranges: 3-5 years and 11 – 15 years old) forbids them to go outside while he works. 11-15year old cares for the 3-5 year old. | | | | Non-slum | Not willing | Male,  Age range: 31- 40 years old | | 1 daughter:  Age range: 3-5 years old | Working as a tailor from 8am to 6pm. Wife works in Saudi Arabia | |
| C-039 | Primary care giver: grandmother of 2 boys one who is at school (age ranges under 3 and 11 – 15 years old) | | | | Slum | Willing | Female,  Age range: over 60 years old | | 1 grandson:  Age range: Under 3 years old | Housewife | |
| C-063 | Primary care-giver: grandmother to girl (age range under 3). Grandfather and parents look after the children in the morning while she works and she cares for them in the afternoon. | | | | Slum | Willing | Female  Age range: over 60 years old | | 1 grand-daughter:  Age range: Under 3 years old | She works as a house maid in the mornings. | |
| Co-Design Focus Group participants purposively sampled from survey respondents willing to use child-care centre | | | | | | | | | | | |
|  |  | | | | Who looks after under-5 when working | | | | Children Under 5yrs | Occupation | |
| FG-P1 | Mother of three children, 2 older sons | | | | Takes under-5 to work with her | | | | 1 daughter | Business | |
| FG-P2 | Mother of two children, 1 older daughter | | | | Work-based centre-based child-care | | | | 1 son | House maid | |
| FG-P3 | Mother of two children, 1 older daughter | | | | Grandparents | | | | 1 daughter | Factory worker | |
| FG-P4 | Mother of three children older daughter, son | | | | Grandmother | | | | 1 daughter | Factory worker | |
| FG-P5 | Mother of one child | | | | Grandmother | | | | 1 son | Service | |
| FG-P6 | Mother of two children, 1 older daughter | | | | Grandmother | | | | 1 daughter | Tailor | |
| FG-P7 | Mother of two children, both under-5 years | | | | Mother’s sister (child’s aunty) | | | | 2 daughters | House maid | |
| Individual interviews with community leaders | | | | | | | | | | | |
| CL_IMAM | Iman of the area, has been resident for over one year | | | | Male | Age range: 41-50 years old | Supportive – wants to include religious education for under-5s | | | | |
| CL_WC | Ward commissioner – elected representative | | | | Male | Age range: 41-50 years old | Not supportive as voters good middle-class not poor working mums | | | | |
| CL-BM | Owner of a small machine-tools factory | | | | Male | Age range: 51-60 years old | Sees need for centre-based child-care for workers where both parents work | | | | |
| CL-HT | Head-teacher – secondary school, has been a teacher in the area over 30 years | | | | Male | Age range: 41-50 years old | Supportive but cautious about level of trust | | | | |
| CL-PS | Social worker and business | | | | Male | Age range: 31-40 years old | Supportive but aware of practical challenges and potential high demand if a quality trusted service | | | | |
| Individual interviews with users, ex-users and staff of the centre-based child-care model | | | | | | | | | | | |
| User 1 | | 2 children – 1 under 5 | | Housewife | | | | Age range:21-30 years old | | | |
| User 2 | | 2 children – 5 and one under 5 | | Works outside the home - unspecified | | | | Unknown | | | |
| User 3 | | 2 children – one under-5 | | Factory worker | | | | Age range:21-30 years old | | | |
| User 4 | | 3 children – one under 5 | | Makes sweets and handicrafts at home | | | | Age range:31-40 years old | | | |
| User 5 | | One child under-5 | | Factory worker | | | | Age range:21-30 years old | | | |
| Ex-user 1 | | One under-5 | | Housewife | | | | Age range:21-30 years old | | | |
| Ex-user 2 | | Two under 5 children | | Housewife | | | | Age range:21-30 years old | | | |
| Ex-user 3 | | One under 5 child | | Domestic help | | | | Age range:21-30 years old | | | |
| Non-user 1 | | One under 5 child | | Government service job | | | | Unknown | | | |
| Non-user 2 | | One under 5 child | | Housewife | | | | Unknown | | | |
| Non-user 3 | | One under 5 child | | Service job | | | | Unknown | | | |
| Support worker 2 | | Bachelor in Social Science | | Assistant centre-based child-care provider | | | | Age range:21-30 years old | | | |
| Centre-based child-care worker 1 | | Undergraduate student | | Main centre-based child-care provider | | | | Age range:21-30 years old | | | |
| Individual interviews with policy-makers | | | | | | | | | | | |
| ID | | | Role | | | | | | | | Sex |
| Policy Maker-01 | | | Senior scientist and ECD Specialist, National NGO and research organisation | | | | | | | | Female |
| Policy Maker-02 | | | Communication officer, International NGO specialising in ECD and provision for under-5s | | | | | | | | Female |
| Policy Maker-03 | | | Director, ECD Network | | | | | | | | Female |
| Policy Maker-04 | | | Project manager,  International NGO specialising in ECD and provision for under-5s | | | | | | | | Female |
| Policy Maker-05 | | | Manager. ECD  International NGO specialising in ECD and provision for under-5s | | | | | | | | Female |

**Table S3: Key features of urban childcare centre model following co-design and shaping during 10 months of implementation**

| **Features** | **Specification** |
| --- | --- |
| Structure of the centre | Two rooms for children’s activities (i.e. ECD stimulations and resting/sleeping) by age group (i.e. under 3 and above 3 years) in a safe, suitable and easily accessible place. |
| Staff: Childcare centre caregiver | Two caregivers for child supervision and ECD activities. Anchal mothers are typically females who are also member of the community where the Anchal is located. This helps to build community trust in the centre. |
| Staff: Assistant (helper) | One assistant for child supervision, cleaning and maintenance |
| Capacity | 25 children per centre (child-caregiver ration= 13:1) |
| Training provided to Staff | Basic Training (5 days training on safety, ECD stimulations, parent engagements, centre management etc.) + First Aid Training (2 days) |
| Day care Fees | None |
| Operation Hours | 8 am to 5 pm considering working mothers (9 am to 3pm during the month of Ramadan) |
| Ensuring safety | Constant supervision by centre staff, signing in and out policies & safe environment. |
| Promoting child nutrition | Parents are educated through monthly meetings and individual discussion on fulfilling the child’s optimum nutrition requirements with locally available products and produce. During childcare centre hours, parents provide lunch and the centre provides food supplements in the form of nutritious snacks (e.g. milk, egg, fruit, nuts). |
| Food management | One kitchen space with fridge and microwave facilities allowed for hygienic food storage and food preparation. |
| Engaging parents | Monthly meetings are arranged to engage parents in problem solving and key decision makings (e.g. how to send the child home from Anchal when the parent is sick). Beside monthly meetings individual communications with parents/guardians are conducted by the centre caregiver as per need. |
| Engaging the community | There is a centre management committee comprising of local school headmaster and willing parent members. Committee are regularly updated about the centre’s activities and the progress of children. It is seen as a way of engaging the community as well as ensuring accountability of the centre to the community.  Before setting up the centre, house to house visits paid by centre caregiver to inform the community about the centre. |

**Table S4: Child-care centre user group meetings**

| **Meeting** | **Participants** | **Issues discussed** | **Solutions tried** |
| --- | --- | --- | --- |
| 1 | 6 mothers  1 grandmother  1 father | 1. Keeping children away from the centre when sick 2. Role of fathers and engagement in user meetings | 1. Explaining the policy 2. Asked to encourage father to come to the centre and meetings |
| 2 | 4 mothers  1 father | 1. Procedure when parents are delayed in picking-up their children 2. Provision of food – challenges of using food brought from home 3. Safety of the children and ensuring they are collected by agreed person 4. Possibility of holding monthly user group meetings | 1. Staff worked late in shifts  2. provision of snacks such as boiled eggs, fruit and bread  3. ID cards for children and guardians approved for collection  4. limited time available to join the group regularly, dropping in/out of users’ group was sufficient |
| 3 | 2 fathers  1 mother  1 grandmother  1 centre-based child-care staff  1 head-teacher as committee member | 1. Charging 100taka for snacks  2. Management of sick children  3. Increase participation of parents in users’ group  4. Challenges of dropping children in the morning | 1. Any payment will be challenging for poorer families – no fees.  2. Agreed parents should keep sick children at home  3. Discuss with parents when dropping/collecting  4. Centre-based child-care staff offered to pick child from home |

**Table S5: The development of meta-inference across the methods used in all phases of the study**

| **Method:**  **Factors driving demand for centre care:** | **Household survey (August 2017)** | **First round of SSIs parents/care-givers** | **Co-design FG, centre users’ meetings, centre records, staff SSIs** | **SSIs second round users, ex-users, non-users** | **Follow-up household survey (6-months)** | **Policy-maker interviews** | **Meta-inference summary** |
| --- | --- | --- | --- | --- | --- | --- | --- |
| i) Perceived child benefits | 24% (95% CI: 16%-37%) reported turning down paid work due to lack of child-care  Higher need for under 3.5 yrs old: AOR 2.9 (1.4-6.2)  Childhood illness associated with those needing a second care-giver (AOR: 1.5, 95% CI: 1.1, 2.2) preferred Madrassas | Parents’ unhappy with limited time available to provide any more than basic care for their child, emphasis on teaching and reading  Value place on religious education | Importance placed by parents on ‘education’ although different perspectives on what this means | Day-care users valued improvements in child development, particularly social, cognitive madrassah popular older children | Importance of free day-care available in Madrasa (11%). Possible explanation for 40% no longer needing care as child over 3.5yrs sent to school or Madrassa | Policy environment increasingly recognising the need for ECD | Education and school readiness a priority  Intuitive understanding of ECD, increasing after using centre, but generally a low priority given to ECD.  High priority given to religious education |
| ii) Social capital and trust in an urban environment | (46% [95% CI: 28%-65%]) fearful of an unknown person caring for their child in day-care | Fear of children going missing; bad influences in urban neighbourhood;  Divisions between long-standing home owners and tenant migrants;  Daughters left alone and forbidden to leave the house;  No evidence of child-care reciprocity between neighbours | User meetings lead to collection ID system developed and engagement with local leaders, household visits and policy on staying late. | Trust develops over time, users and ex-users convinced of safety, but still a concern driving non-use. | 28% untraceable by phone/  home-visit and 41% moved home showing transience of urban families |  | Concern for children picking up 'bad' language and behaviour  Fear of abduction and abuse  Time needed to build trust in child-care centre safety and staff |
| iii) Family first | 86% of primary care givers wert mothers, 6% grandmothers; no unrelated neighbours. | Day-care as last resort: female family members preferred  Siblings (boys and girls) providing child-care.  Parents concerned that grandparents/other relatives only providing basic care |  | Some initial resistance from husbands noted, but overcame with positive experiences of the centre. Where child has a long-term condition or problem settling, they are removed from centre. | Possible explanation for 40% no longer needing care; although only 3% said husband forbade centre use. |  | Gender norm for mothers to stay home to care for the family  if mother not available, preference for female relatives or older siblings (boys or girls) to care for under-5s, Child-care centres seen as last resort. |
| iv) Demand: work and childcare | 84% would use child-care centre, slum households more likely to need child-care (AOR: 3.8, 95% CI: 1.4, 10)  83% ‘housewives: not working outside the home’. | Need is greatest for working parents, particularly working mothers  Child-care options included taking child to work, leaving alone at home, siblings, grandparents. | Seasonal demand – poorer tenants likely to return to rural areas particularly during Ramadan.  Payment – many unable to pay.  Some require care into the evening | Benefits of being able to work  Day-care enabled women to generate income at home as well as outside. | Only 14% reported needing day-care | The need is great, but only free day-care will attract urban poor. | Parents, particularly mothers, need to work long-hours outside the home and struggle to pay-for care  Demand may be seasonal  Hidden demand from women working in the home |
| v) Feasibility - fees, food, hours and engagement | Require centre near to home  Prepared to pay mean of 283 taka/month and provide food, provision for under 3.5years important | Prepared to bring their own food  Hours 8-5pm  Parents disagree over background and training of staff | Limited enrolment and fluctuating demand  Participation in user-group challenging  Any payment a challenge for low-income families  Provision for late-stay  Clear policy/ practice on safety  Provision of some food vital for equity | Positive responses from users and ex-users, no complaints on fees, food, practices. |  |  | Those most in need struggle to pay any fees, sustainability a major issue  some need care into the evening and provision of food a requirement, parental engagement challenging |
