# Supplementary material for: Understanding demand for, and feasibility of, centre-based child-care for poor urban households: a mixed methods study in Dhaka, Bangladesh
Source: BMC Public Health. 2020 Dec 10;20:1899. doi: 10.1186/s12889-020-09891-z (PMC7727228; doi:10.1186/s12889-020-09891-z)
Supplement: Supplementary file 2 — Additional file 2. Anchal Childcare centre demand questionnaire English. [file 12889_2020_9891_MOESM2_ESM.docx]

**Sustainable child day-care for 1-4 year olds in disadvantaged urban communities in Dhaka, Bangladesh**

**CIPRB, House # B-162, Road # 23, New DOHS, Mohakhali, Dhaka- 1206, Bangladesh**

**House ID No.:**

**ID of HH head:**

**ID of Respondent:**

**Date of interview:**

**Start time:**

**End time:**

**Interviewer’s Name:**

**Index Child’s Age: 1 to < 3 yr 3 to < 5 yr**

**Durable housing of a permanent nature that protects against extreme climate conditions: Yes No**

**Security of tenure that prevents forced evictions: Yes No**

**Sufficient living space which means not more than three people sharing the same room: Yes No**

**Easy access to safe water in sufficient amounts at an affordable price: Yes No**

**Access to adequate sanitation in the form of a private or public toilet shared by a reasonable number of people.: Yes No**

**HH status: Slum Non-slum**

**Section A:**

| A1.  ID | A2.  Age in years | A3.  Sex | A4.  Religion | A5.  Educational years | A6.  Occupation | A7.  Monthly income (tk) | A8.  Relationship with index child |
| --- | --- | --- | --- | --- | --- | --- | --- |
| 1 |  |  |  |  |  |  |  |
| 2 |  |  |  |  |  |  |  |
| 3 |  |  |  |  |  |  |  |
| 4 |  |  |  |  |  |  |  |
| 5 |  |  |  |  |  |  |  |
| 6 |  |  |  |  |  |  |  |
| 7 |  |  |  |  |  |  |  |
| 8 |  |  |  |  |  |  |  |
| 9 |  |  |  |  |  |  |  |
| 10 |  |  |  |  |  |  |  |

**Codes for Section A:**

**সেকশন A এর কোডঃ**

**Sex:** 1. Male, 2. Female, 3. Transgender 97. Not known, 98. Refused

**Religion:** 1. Islam, 2. Hinduism, 3. Christianity, 4. Buddhism, 97. Not known, 98. Refused, 99. Others, specify___________________

**Occupation**: 1. Garments worker 2. Works in plastic bag factory, 3. Works in recycling industry, 4. Maid/servant, 5. Business, 6. Student, 7. Government Service, 8. Private service, 9. Housewife, 10. Work from home, 11. Retired, 12. Unemployed, 13. Unable to work, 14. Beggar, 97. Not known, 98. Refused, 99. Others, specify ___________-

**Relationship with index child:** 1. Mother, 2. Father, 3. Sister, 4. Brother, 5. Grandmother, 6. Grandfather, 7. Uncle, 8. Aunt, 9. Cousin, 88. Neighbor, 97. Not known, 98. Refused, 99. Others, specify__________________

| A9. Index child’s Unique ID |  |
| --- | --- |
| A10. Index child’s Height (in cm) |  |
| A11. Index child’s weight (in kg) |  |

| **B. Children’s Injury Information** | | | | | |
| --- | --- | --- | --- | --- | --- |
| Under 5 child injury information (last 6 months)  Child injury is defined as if the child suffered by any of the following mechanisms; Drowning, Road Traffic Injuries (RTI), Burns, Falls, Cut injuries, Injuries due to falling object, Machine injury, Unintentional poisoning, Animal injury, Electrocution, Suffocation, Violence, Suicide; and sought medical care or could not do their regular activities (playing, feeding, talking, walking) for at least one-day during the last three months | | | | | |
| B1. U-5 child’s ID | B2. Any Injury in last 6 months | B3. Mechanism of injury | B4. Place of injury | B5. Type of injury | B6. Who was looking after child at time of injury (Child’s relation with that person) |
|  |  |  |  |  |  |
|  |  |  |  |  |  |
|  |  |  |  |  |  |
|  |  |  |  |  |  |
|  |  |  |  |  |  |

**Codes for Section B:**

**Any Injury in last 6 months:** 1. Yes, 2. No, 97. Not known, 98. Refused

**Mechanism of injury**: 1. Non-fatal Drowning, 2. Road Traffic Injuries (RTI), 3. Burns, 4. Falls, 5. Cut injuries, 6. Injuries by blunt object, 7. Machine injury, 8. Unintentional poisoning, 9. Animal bite/ attack, 10. Electrocution, 11. Suffocation, 12. Violence, 97. Not known, 98. Refused, 99. Others, specify__________

**Place of injury:** 1. Own house, 2. Neighbor or friend’s house, 3. Caregiver’s house, 4. Playground, 5. Road/highway, 6. Footpath, 7. Railway station/Ferry/ Launch station, 8. Agricultural field, 9. Industry/factory/workshop, 10. Water reservoir 11. Medical service area, 12. Daycare centre, 13. River, 14. Other water source, 97. Not known, 98. Refused, 99. Others, specify___________

**Type of injury:** 1. Violence/assault, 2. Non- intentional, 97. Not known, 98. Refused

**Who was looking after child at time of injury:** 1. Mother, 2. Father, 3. Sister, 4. Brother, 5. Grandmother, 6. Grandfather, 7. Uncle, 8. Aunt, 9. Cousin, 10. Non-family member, 11. Paid caregiver, 97. Not known, 98. Refused, 99. Others, specify____________________________

| C. **Communicable disease:** Did under five children of your household suffer from any of the following in last 1 month- | | | | | |
| --- | --- | --- | --- | --- | --- |
| C1.  U-5 child’s ID | C2.  Fever | C3.  Cough and Cold | C4.  Diarrhoea and /or vomiting (repeated) | C5.  jaundice (yellow discoloration of eyes or skin) | C6.  skin rash along with fever and cold |
|  |  |  |  |  |  |
|  |  |  |  |  |  |
|  |  |  |  |  |  |
|  |  |  |  |  |  |
|  |  |  |  |  |  |

**Codes for Section C:**

**Fever:** 1. Yes, 2. No, 97. Not known, 98. Refused

**Cough and Cold:** 1. Yes, 2. No, 97. Not known, 98. Refused

**Diarrhoea and /or vomiting (repeated):** 1. Yes, 2. No, 97. Not known, 98. Refused

**Jaundice (yellow discoloration of eyes or skin):** 1. Yes, 2. No, 97. Not known, 98. Refused

**Skin rash along with fever and cold:** 1. Yes, 2. No, 97. Not known, 98. Refused

| **D. Caregiver of under five children** | | | | | | | | |  |
| --- | --- | --- | --- | --- | --- | --- | --- | --- | --- |
| D1.  U-5 child’s ID | D2.  ID of Primary Caregiver | D3.  Relation of primary caregiver with child | D4.  Works outside | D5.  Working days of week | D6.  Working hours of day | D7.  Missed a day’s work in last month due to taking care of u-5 | D8.  Ever not been able to take up a job due to taking care of u-5 | D9.  Need of secondary caregiver | D10.  Relation of secondary caregiver with child |
|  |  |  |  |  |  |  |  |  |  |
|  |  |  |  |  |  |  |  |  |  |
|  |  |  |  |  |  |  |  |  |  |
|  |  |  |  |  |  |  |  |  |  |
|  |  |  |  |  |  |  |  |  |  |

**Codes for section D**:

**Relation of primary caregiver with child:** 1. Mother, 2. Father, 3. Sister, 4. Brother, 5. Grandmother, 6. Grandfather, 7. Uncle, 8. Aunt, 9. Cousin, 88. Neighbor, 97. Not known, 98. Refused, 99. Others, specify_________________________________________

**Works outside:** 1. Yes, 2. No, 97. Not known, 98. Refused

**Missed a day’s work in last month due to taking care of u-5:** 1. Yes, 2. No, 97. Not known, 98. Refused

**Ever not been able to take up a job due to taking care of u-5:** 1. Yes, 2. No, 97. Not known, 98. Refused

**Child needs any paid caregiver:** 1. Yes-paid, 2. Yes-unpaid, 3. No, 97. Not known, 98. Refused

| **E. Need for day-care:** | **Code/Response**  **কোড/উত্তর** |
| --- | --- |
| E1. For how long are you living in this area?  1. Less than 1 year, 2. At least 1-2 years, 3. 3 or more years, 97. Not known, 98. Refused |  |
| E2. For how long do you plan to live in this area?  1. Less than 1 year, 2. At least 1-2 years, 3. 3 or more years, 97. Not known, 98. Refused |  |
| E3. Do you feel the need of a quality day-care center for the u-5 child/children of this household? (if no, skip to next section)  1. Yes, 2. No, 97. Not known, 98. Refused |  |
| E4. Does ________ (name if index child) needs supervision from another person?  1. Yes, 2. No, 97. Not known, 98. Refused |  |
| E5. If yes, During which days of the week _______ (name if index child) needs supervision from another person?  Ans. |  |
| E6. During which time of the day _______ (name if index child) needs supervision from another person, if applicable?  Ans. |  |
| E7. Would you send any of your under-5 children in this household to day-care?  1. Yes, 2. No, 97. Not known, 98. Refused |  |
| E8. If not, What is the cause?  1. Problem of accepting of a stranger to look after the child, 2. Having other children belonging to the lower socio-economic condition in the day care, 3. Decision makers of the family will not allow, 4. Nutrition of the child will be hampered, 97. Not known, 98. Refused, 99. Others, specify___________________________ |  |
| E9. How many of the under 5 children in this household can be sent to day-care? (97. Not known, 98. Refused) |  |
| E10. At what age would you feel comfortable sending your child to day care? (97. Not known, 98. Refused) |  |
| E11. during which days of the week _______ (name if index child) can be sent to daycare? |  |
| E12. And, During which time of the day _______ (name if index child) can be sent to daycare? |  |
| E13. At what location you want the day-care to be placed?  1. Near home, 2. Near workplace, 3. Near home or workplace, 97. Not known, 98. Refused, 99. Others, specify ________________________________________________________________________ |  |
| E14. Would you provide dry or cooked food for your child in day-care?  1. Yes, 2. No, 97. Not known, 98. Refused |  |
| E15. How much money in taka can be be paid per day as fees for daycare service for each child if food was sent from home? |  |
| E16. How much money in taka can be be paid per day as fees for daycare service for _______ (name if index child) if food was provided by daycare center? |  |
| E17. Would you be prepared to pay a higher fee for your child to attend day care in order to subsidize poorer children?  1. Yes, 2. No, 97. Not known, 98. Refused |  |
| E18. Will it be acceptable if ______ (name if index child) is kept in the same room of day care center belonging to a less privileged/poorer family/lower socio-economic group?  1. Yes, 2. No, 97. Not known, 98. Refused |  |
| E19. Would the primary caregiver (name) of _______ (name if index child) join a parents committee to have a say, along with other parents, in how the center was run if _______ (name if index child) went there?  1. Yes, 2. No, 97. Not known, 98. Refused |  |

| **F. Feasibility of day-care** | **Code/Response**  **কোড/উত্তর** |
| --- | --- |
| F1. Do you think people in this community would use child day care for their children if they were able to afford it?  1. Yes, 2. No, 97. Not known, 98. Refused |  |
| F2. If yes, who might use the day care in your community? (multiple answers will be accepted)  1. Families with working mother, 2. Single mother, 3. Long term ill household member, 4. Child with no mother, 5. Child with no parents, 6. Families with good income, 7. Families with poor income, 8. Educated families, 97. Not known, 98. Refused, 99. Others, specify___________ |  |
| F3. What do you think will be the three important challenges to establish a day-care (like, anchal) in this community? (multiple answers will be accepted)  1. Money/fees for day care, 2. Accepting of a stranger to look after the child, 3. Having other children belonging to the lower socio-economic condition in the day care, 4. Mobile population, 5. Elders of the family will not allow, 6. Nutrition of the child will be hampered, 7. No challenges, 97. Not known, 98. Refused, 99. Others, specify___________________________ |  |

| **G. Identifying community leaders** | **Codes/Response**  **কোড/উত্তর** |
| --- | --- |
| G1. According to your understanding who are the community leaders in your community? (multiple answers will be accepted)  1. Ward commissioner, 2. School headmaster, 3. School teacher, 4. Imam (religious leader), 97. Not known, 98. Refused, 99. Others, specify___________________ |  |

| **H. Caregivers of children for other HH** | **Codes/Response**  **কোড/উত্তর** |
| --- | --- |
| H1. Does anyone in the household looks after any under-five child from another household?  1. Yes, 2. No, 97. Not known, 98. Refused |  |
| H2. If yes, Who is that person? (put person’s ID from section A) |  |
| H3. Whose children (under five) does she look after?  H3. তিনি কার বাচ্চা দেখাশোনা করেন?  1. Of a relative, 2. Of a neighbor, 97. Not known, 98. Refused, 99. Others, specify_____________________________________  1. আত্মীয় এর , 2. প্রতিবেশীর, 3. জানা নেই, 98. উত্তর দানে অস্বীকৃতি, 99. অন্যান্য, উল্লেখ করুন_____________ |  |
| H4. How old are the children?  Ans |  |
| H5. How often does she look after them?  Ans. |  |
| H6. For how long does she look after them?  Ans. |  |
